# Bacterial Tracheitis: A New Presentation of a Well-Known Disease

**DOI:** 10.7759/cureus.63697

**Published:** 2024-07-02

**Authors:** Mafalda Moreira, Paula Regina Ferreira, Alzira Sarmento, Ana Lúcia Cardoso

**Affiliations:** 1 Pediatrics and Child Health, Centro Hospitalar do Tâmega e Sousa, Penafiel, PRT; 2 Pediatric Intensive Care Unit, Centro Materno Infantil do Norte, Centro Hospitalar Universitário de Santo António, Porto, PRT; 3 Pediatric Intensive Care Unit, Centro Materno Infantil do Norte, Centro Hospitalar Universitário do Porto, Porto, PRT

**Keywords:** pediatric emergency medicine, group a streptococcus pyogenes, steeple sign, acute airway obstruction, bacterial tracheitis

## Abstract

Bacterial tracheitis (BT) is an uncommon life-threatening condition that results in acute upper airway obstruction. Classical signs include a toxic appearance, stridor, tachypnoea, and fever, often leading to rapid clinical deterioration. Recent studies have shown a shift in BT epidemiology and presentation, where stridor and respiratory distress are now predominant. A poor response to corticosteroids or nebulized epinephrine is also commonly described, along with a need for mechanical ventilation. We present the case of a five-year-old boy admitted to the emergency department with cough, stridor, and dyspnea that had significantly worsened over the previous hours. He presented reasonable general condition, marked retractions, poor air entry, stridor, and wheezing. Investigation revealed a slight elevation of C-reactive protein and leukocytosis with neutrophilia. Anteroposterior x-ray showed narrowing of subglottic airways (steeple sign). There was no response to oral/nebulized corticoids, nebulized adrenaline, or bag-valve-mask oxygenation. Antibiotic therapy with ceftriaxone was initiated. Due to deteriorating clinical conditions with severe respiratory acidosis, orotracheal intubation was required. Later *Streptococcus pyogenes* was isolated in the bronchial secretions and a targeted antibiotic regimen was administered. Progressive clinical and analytical improvement was observed with no complications. Although uncommon, BT remains a severe infectious condition affecting otherwise healthy children. Our case underscores the severity of the disease and the imperative for invasive interventions to achieve favorable outcomes. It also supports recent findings indicating a shift in predominant symptoms and prognosis. Clinicians must be vigilant and knowledgeable, recognizing that worsening stridor and respiratory distress unresponsive to conservative treatment are key indicators for diagnosing BT.

## Introduction

Bacterial tracheitis (BT), also known as membranous laryngotracheobronchitis, is an uncommon life-threatening condition that results in acute upper airway obstruction. Most severe cases may progress with respiratory distress, cardiac arrest, and death [1-3]. As a consequence of their smaller airways and narrow subglottic region (the narrowest portion of the upper airways in childhood, as opposed to the glottis in adulthood), children are more frequently affected than adults [4].

Studies have shown that the estimated incidence of BT in children is 0.1 per 100000. It occurs more frequently during autumn and winter months, coinciding with seasonal epidemics of the main respiratory viruses, such as influenza, parainfluenza, respiratory syncytial virus, and rhinovirus [3-5]. Mortality rates are estimated at around 20% [6].

The classic description of a child with BT is a patient with a toxic appearance, stridor, tachypnea, voice changes or hoarseness, fever, and dysphagia that rapidly deteriorates within a period of two to 10 hours [1,2,6,7] Many patients present prodromal symptoms, such as low-grade fever, cough, and rhinorrhea. This clinical evolution supports the hypothesis that BT is a bacterial infection superimposed over a viral upper respiratory tract infection [1,3,6]. The most frequently identified bacteria are methicillin-sensitive Staphylococcus aureus (MSSA), Hemophilus influenzae, Moraxella catarrhalis, and Streptococcus pneumoniae [1,2].

The main differential diagnoses that must be considered when a child presents with stridor and respiratory distress are croup, BT, and epiglottitis. Croup is a viral illness characterized by subglottic swelling and a barking cough that mostly occurs in children under six years old [2,4]. In some cases, BT appears to be a complication of croup. In cases of croup, children present a favorable evolution with steroids or nebulized epinephrine so the diagnosis of BT must be suspected when there is a marked worsening of the clinical course or poor response to this treatment. On the other hand, epiglottitis consists of an inflammation of the epiglottis, primarily caused by infection. Children may present high fever, drooling, distress, dysphonia, dysphagia, and refusal to lie down [4]. The main differences between BT and epiglottitis are the less severe and acute presentation of the first one and the presence of enlarged epiglottitis or thumb sign on the X-ray in the cases of epiglottitis [5]. Bronchoscopy, which is a procedure that allows direct visualization of the tracheobronchial tree and, in some cases, the performance of treatment, should be considered case-by-case. It depends on the child’s age and severity of symptoms, being important to confirm the diagnosis of BT and exclude epiglottitis. [4,5].

Airway management is a crucial part of treatment, being intubation/emergent tracheostomy required in up to 80% of cases [1,4,6]. Systemic antibiotic therapy must be initiated empirically, taking into account the most frequently isolated bacteria.

Over the past years, several studies have been reporting a shift in BT epidemiology and presentation, with patients presenting milder symptoms and no signs of systemic infection, such as high fever or toxic appearance [1-3]. Therefore, BT should be suspected in cases of stridor and respiratory distress, with poor or absent response to systemic corticosteroids or nebulized epinephrine [7]. The presence of purulent secretions, tracheal inflammation, and subglottic narrowing with a normal epiglottis on direct larynx observation confirms the diagnosis [4].

Although this new presentation is normally associated with less severe outcomes, clinicians must be aware of these signs and symptoms, in order not to underestimate the clinical findings and act promptly [1].

## Case presentation

A five-year-old boy was admitted to the pediatric emergency department (ED) with cough, stridor, and dyspnea with significant deterioration over the previous two hours. The family history was unremarkable and he had a personal history of epilepsy, treated with sodium valproate. The national vaccination program was up to date with complete vaccination against Haemophilus influenzae.

The first symptoms started a week before and consisted of fever and irritating cough, with a favorable response to oral corticosteroid therapy and nebulized adrenaline.

Upon arrival at the ED, the patient's overall condition was reasonable, with no apparent signs of toxicity. Physical examination revealed marked retractions, poor air entry, inspiratory stridor, and wheezing. The respiratory rate was 29 breaths per minute, the oxygen saturation was 98%, and the heart rate was 140 beats per minute. To address the presenting respiratory distress, a therapeutic regimen consisting of oral glucocorticoids, nebulized adrenaline, and nebulized budesonide was initiated.

Due to the presentation of severe airway obstruction, characterized by generalized marked retractions, poor air entry, and fatigue, immediate intervention was required to provide adequate oxygenation. A bag-valve mask with 100% oxygen was initiated, which was subsequently optimized with non-invasive ventilation to improve air exchange. In light of the suspicion of BT (no response to 10 mg of prednisolone, 5 mL of nebulized epinephrine, and 1 mg of nebulized budesonide), the patient was initiated on broad-spectrum antibiotic therapy with ceftriaxone.

**Figure 1:**
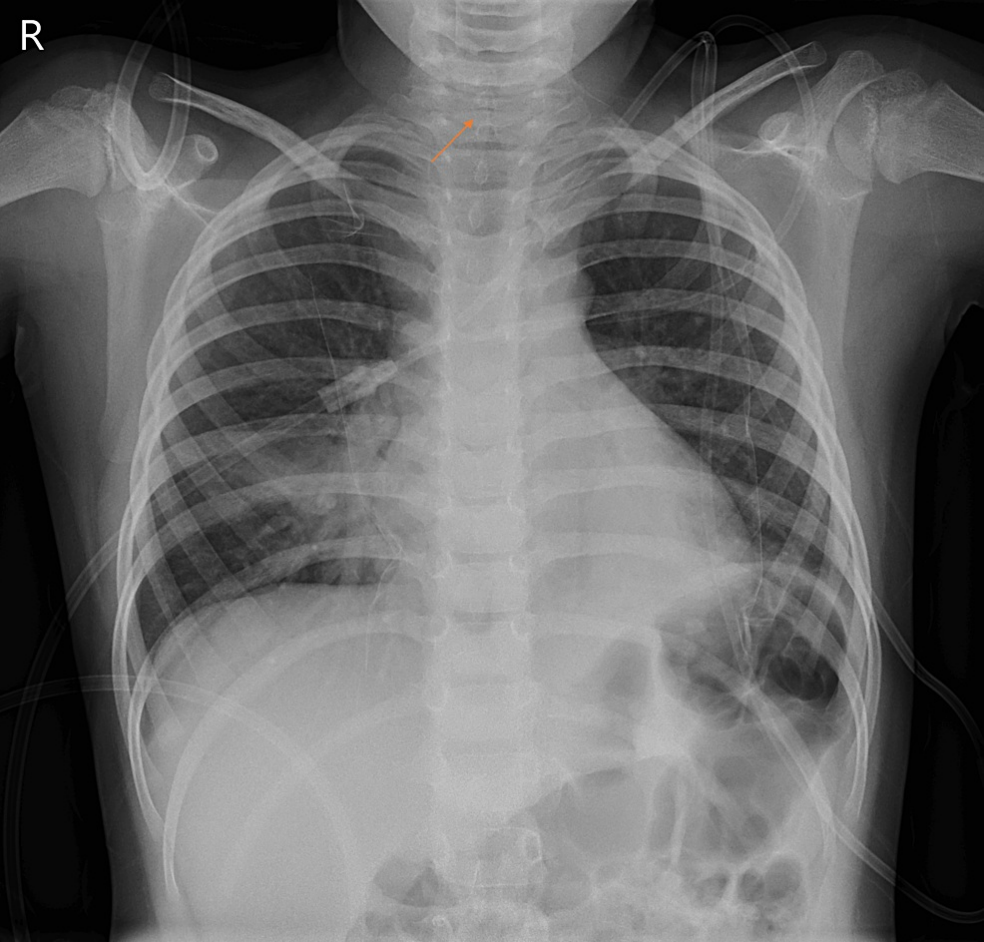
Anteroposterior x-ray showing narrowing of subglottic airways, known as steeple sign

The initial investigation revealed a slight elevation of C-reactive protein and leukocytosis with neutrophilia (Table 1). The anteroposterior radiograph showed distension of the lower pharyngeal region and narrowing of the subglottic airways, exhibiting the classical steeple sign (Figure 1). A bronchoscopy was not performed. Blood culture was negative and adenovirus and coronavirus were identified on the nasopharyngeal wash and Streptococcus pyogenes was isolated in the bronchial secretions.

**Table 1:** Laboratory investigation

	Results	Reference range
Hemoglobin (g/dL)	12	11.5-15
Leukocytes (x10^3 ^µL)	22.93	5-14.5
Neutrophils (x10^3 ^µL)	17.95	1.5-8
Lymphocytes (x10^3 ^µL)	1.47	1.5-7
Platelets (x10^3 ^µL)	335	150-400
C-reactive protein (mg/L)	38.5	0-5

Despite previously described interventions, the patient experienced a continued clinical deterioration, and blood gas analysis revealed severe respiratory acidosis (pH: 7.19 and pCO_2_: 76.1). As a result, it was decided that orotracheal intubation was necessary, and the patient was subsequently admitted to an intensive care unit for further management. Given that intubation can be challenging in cases of narrowed or obstructed subglottic airways, an anesthesiologist was consulted to provide assistance during the procedure, in accordance with current literature recommendations.

As soon as Streptococcus pyogenes’ susceptibility test proved the sensitivity to Penicillin and Clindamycin, the regimen was tailored appropriately and continued for a total of 10 days. Clindamycin was used for its action as an inhibitor of bacterial protein synthesis. The child was extubated on day 5 and needed high-flow nasal cannula therapy for two days with a maximum fraction of inspired oxygen of 0.45. Dexamethasone was maintained for seven days.

Our patient presented progressive clinical and analytical improvement and was discharged to the ward on day 8, spontaneously ventilating with a flow rate of oxygen supplementation of 1 liter/minute. There were no short- or long-term complications.

## Discussion

BT, though uncommon, can lead to infectious upper airway obstruction, and its clinical and epidemiological characteristics appear to be evolving [1,2]. Nowadays, children rarely exhibit the classic toxic appearance and high fever. Instead, as illustrated in our case, they display stridor and rapidly worsening respiratory distress (manifested by marked retractions, poor air entry, and fatigue), with poor or no response to systemic corticosteroid or nebulized epinephrine. In this case, the continuous clinical deterioration with severe respiratory acidosis warranted the need for orotracheal intubation and admission to the intensive care unit. This is consistent with the literature, where intubation is required in the majority of cases. Several studies, such as the one conducted by Casazza et al., support our findings, underscoring the importance of recognizing the failure to respond to conservative treatment as a hallmark of BT in cases of upper airway obstruction [1,2].

The incidence of BT has decreased significantly with the vaccination against Haemophilus influenzae, which is included in our national vaccination program and readily available for all citizens [3,4].

The determination of the agent responsible for BT is essential to optimize treatment, which should involve not only mechanical ventilation but also targeted therapy for the causative agent. The most frequent agent of BT is MSSA, followed by Haemophilus influenzae, Moraxella catarrhalis, and Streptococcus pneumoniae [1,2]. Streptococcus pyogenes, the causative agent in our case, is not one of the main bacteria found, being reported in approximately 2-6% of the cases [1-3]. The recommended antibiotic regimen consists of Ampicillin and Clindamycin, administered for 7-10 days, that was initiated in our case as soon as the sensitivity of Streptococcus pyogenes was known.

Although the presentation can be alarming, most children with BT recover without sequelae when treatment is promptly initiated.

## Conclusions

BT presentation has evolved over the past years, and the classical toxic appearance characterized by high fever, respiratory distress, and dysphagia is rarely encountered. Instead, patients exhibit signs of respiratory distress that rapidly worsens. Clinicians need to be vigilant regarding this new presentation of BT, as the majority of patients may require intensive care support and intubation within a short timeframe, often necessitating mechanical ventilation for a brief period. Given the good prognosis for children who receive attempted treatment and ventilatory support, a correct diagnosis is essential in these cases.
